# Prediction and forewarning of axial force of steel bracing in foundation pit based on verhulst model

**DOI:** 10.1371/journal.pone.0265845

**Published:** 2022-03-25

**Authors:** Huan Chen, Ke Zhang, Yibo Jiang, Zheng Shi

**Affiliations:** 1 College of Business, Hohai University, Nanjing, China; 2 Jiangsu Huaiyin Water Conservancy Construction Co., Ltd., Huaiyin, China; Al Mansour University College-Baghdad-Iraq, IRAQ

## Abstract

The axial force of steel bracing is one of the essential indexes to measure the stability of the bracing system of a foundation pit. The steel bracing system of a foundation pit in Ningbo City, China was taken as the research object to guarantee the stability of the steel bracing system of the foundation pit. Besides, the change of axial force between the two steel bracing structures was analyzed to predict the axial force data of the steel bracing and perform the safety forewarning of the steel bracing system. Firstly, GM (1,1) and Verhulst models in the gray model were selected for prediction based on the characteristics of poor information and the small sample size of original monitoring data of the steel bracing. Secondly, the precision of the GM (1,1) model and Verhulst model was compared to determine a more accurate prediction method. Finally, the safety forewarning model of the confidence interval estimation method was established based on the data obtained from the prediction model and the deformation characteristics and indexes of the steel bracing. With the significance levels α = 5% and α = 2% as the demarcating points, the forewarning grades of the steel bracing system of the deep foundation pit were divided, and then the operating state of the current steel bracing system was determined. The results demonstrated that the Verhulst model had better prediction precision compared with the ordinary GM (1, 1) model. Besides, the steel bracing system was in the safe operation range, and the judgment results of the model were consistent with the actual situation of the foundation pit of the steel bracing system. Thus, the Verhulst prediction model and the confidence interval security early forewarning model could be used to judge the stability of the steel bracing system.

## 1. Introduction

With the continuous development of the international community, the utilization of underground space presents a rapid growth trend. In the process of engineering construction, engineering safety accidents cannot be totally avoided, and deep foundation pit collapse accidents account for a large proportion. Foundation pit collapse will not only cause casualties and huge economic losses but also bring an impact on social development [1]. The recent foundation pit collapse is taken as an example. On April 10, 2019, a foundation pit collapsed at a construction site of a farmers’ settlement community in Guangling District, Yangzhou City, Jiangsu Province, killing five people and injuring another. On June 8, 2019, a road cracked near Zhuling Overpass along Dongge Road extension line in Nanning city, Guangxi Province. The cracked area was about 60 meters long and 15 meters wide, and the collapse volume was about 4,500 cubic meters. On September 26, 2019, the foundation pit slope collapsed during the foundation pouring operation of Building 4, at the construction site of Wansheng Homeland, 745 Tianlong South Third Road, Tianhui Town Street, Jinniu District, resulting in the death of 3 workers in the steel binding team. The monitoring of the foundation pit is of great significance for judging the overall stability of the foundation pit, organizing construction, and ensuring construction safety. The first bracing in the foundation pit envelope is generally concrete bracing, and the second and third bracing are steel bracing. This structure can not only enhance the tensile capacity of the joints and avoid the grounding wall kick accident but also prevent the progressive collapse caused by the failure of single bracing [2]. The axial force of steel bracing is an essential safety index, which reflects the stability and development trend of the bracing system of the foundation pit and can measure whether the foundation pit is in a safe state.

In the excavation process of steel bracing, the larger the excavation depth, the longer the exposure time. The axial force will increase with the advancement of engineering construction. This is because during deep excavation, changes in the stress state of the groundmass around the excavation, as well as subsequent ground losses, are unavoidable [3]. After the next bracing is set up, much of the stress will be transferred to the next support beam. Since the axial force of this bracing will become small and stable, timely setting up the next bracing in a certain period of time is conducive to the stability of the bracing system [4, 5]. The delay in the erection of steel bracing will significantly increase the settlement and deformation of the ground envelope structure, producing a negative impact on the foundation pit and the surrounding environment [6, 7]. Therefore, it is necessary to perform a forecast and safety forewarning of the steel bracing after erection.

Among the prediction methods, machine learning methods such as SVM, XGBOOST, and Random Forest are mostly data-driven. Besides, neural network algorithms and other prediction methods also have similar characteristics, such as Akan’s study [8, 9]. The axial force data of steel bracing of foundation pit is generally predicted based on BP neural network. However, the BP neural network needs to consider too much and multifarious data due to the highly complex and nonlinear characteristics of the data. However, not all the research objects have a large amount of data and a large number of data dimensions, especially for engineering construction sites with a complex environment. Thus, it is difficult to reach such high standards. First, the practice has verified that equipment failure rate generally has a certain functional relationship with time, which is called the “bathtub curve” [10, 11]. Additionally, the accumulated initial data may not achieve the amount of data predicted by the neural network because of the short erection time of some steel bracing. Given this situation, the model with the characteristic that “small sample can be predicted” should be selected, namely, the gray prediction model. Little initial data can be used, and the rest of the influence factors can be regarded as gray quantity [12]. In the case of significant trends (the axial force data has a significant increase trend after the erection of steel bracing), the prediction data is more accurate. In recent years, many researchers have employed the prediction model to predict the monitoring data of the foundation pit. Guo et al. [13] predicted the deformation of the foundation pit based on the multivariable MGM (1, m) coupling system model following the principle of self-recall. Akan et al. [14] established a model predicting the unconfined compressive strength of jet grout columns by using multiple linear regression analyses to overcome this problem. Li et al. [15] improved the dynamic gray GM (1,1) model to predict the monitoring data of the foundation pit deformation and achieved higher accuracy and better adaptability. Taken together, the existing research on forecast model has more mature, and through some basic forecasting model, spawned a variety of ways of a combination forecasting model, on the prediction precision and applicable range had great progress, but in the process of practical application, or need to judge the data characteristic of forecasting object, analyzing the characteristics of the object, Choose a style that fits the occasion. When predicting the data of steel bracing systems, it is necessary to take into account the low monitoring frequency of steel bracing system data and relatively few sample data. The targeted selection of the gray prediction model can well fit the change law of the steel bracing system.

GM (1,1) model and Verhulst model are widely used in the prediction of foundation pit monitoring. The GM (1,1) model is primarily adopted to analyze the data with significant exponential law or similar filtering; the Verhulst model focuses on a process of presenting saturation state. HAN et al. adopted the intelligent back analysis method of the gray Verhulst model (GVM) to effectively determine the design parameters and stability of roadways and stopes [16]. HE et al. established a cloud-Verhulst hybrid prediction model by combining a cloud model with the Verhulst model, and this model achieved higher prediction accuracy compared to the traditional statistical model [17]. LEE et al. established a deep foundation pit risk management system based on BIM-3DGIS framework and optimized gray Verhulst model [18]. According to the characteristics of the steel bracing of the foundation pit, the axial force of the steel bracing of the whole foundation pit exhibits an “S” shaped curve, consistent with the characteristics of the Verhulst model; the traditional GM (1,1) model will produce large errors [19]. For example, Zhang et al. [20] established an optimized gray discrete Verhulst model for the prediction of foundation pit settlement and acquired the results with higher accuracy.

The object of this study is the steel bracing system for No.5-4 deep foundation pit in the transfer station of Ningbo Metro Lines 4 and 5. The main body is an envelope structure with underground continuous walls and internal bracing, in which the first and fifth bracing are concrete, and the rest are steel bracing. At present, gray prediction is broadly employed to predict foundation pit settlement. Moreover, it can be replaced, though the settlement monitoring sensor fails, with little impact on the actual project. Nevertheless, the steel strut axial force sensor is a one-time consumable. Hence, the axial force monitoring point of the steel bracing here will be permanently invalid if there is artificial damage in the construction process, or the sensor itself fails. To sum up, it is urgent to conduct the prediction and safety forewarning of the axial force of the steel bracing. This would be helpful to predict the steel bracing data under the failure state of the axial force monitoring sensor of the steel bracing, determine the current stable state of the steel bracing, and judge the monitoring personnel, so as to consider the countermeasures in time.

## 2. Project overview and monitoring results analysis

### 2.1 Project overview

Ningbo Rail Transit Line 4 is a vital radial line from northwest to southeast of the line network, with its starting and terminating stations at CichengStation and Dongqianhu Station, respectively. The overall length of the line is 35.95km, including 24.45km of underground line, 11.2km of elevated line, and 0.3km of transitional section. The whole line involves 25 stations (including 6 transfer stations), composed of18 underground stations and 7 elevated stations, with an average station spacing of 1.49km. It also includes CichengParking Lot and Dongqian Lake Depot.

South Higher Education Park Station is the 22nd station of Ningbo Rail Transit Line 4. The 10th station of the first phase of Line 5 is the T-type transfer island station of Ningbo Rail Transit Lines 4 and 5, located at the intersection of Ningheng Highway and Yinxian Avenue in Yinzhou District. The station of Line 4 is two underground floors, with a double-column three-span box structure and a station scale of 486.5m×20.3m(inside net size). It is located at the south side of the intersection and in the range of Ningheng Highway and the west green belt from north to south.

### 2.2 Installation of axial dynamometer

**Fig 1:**
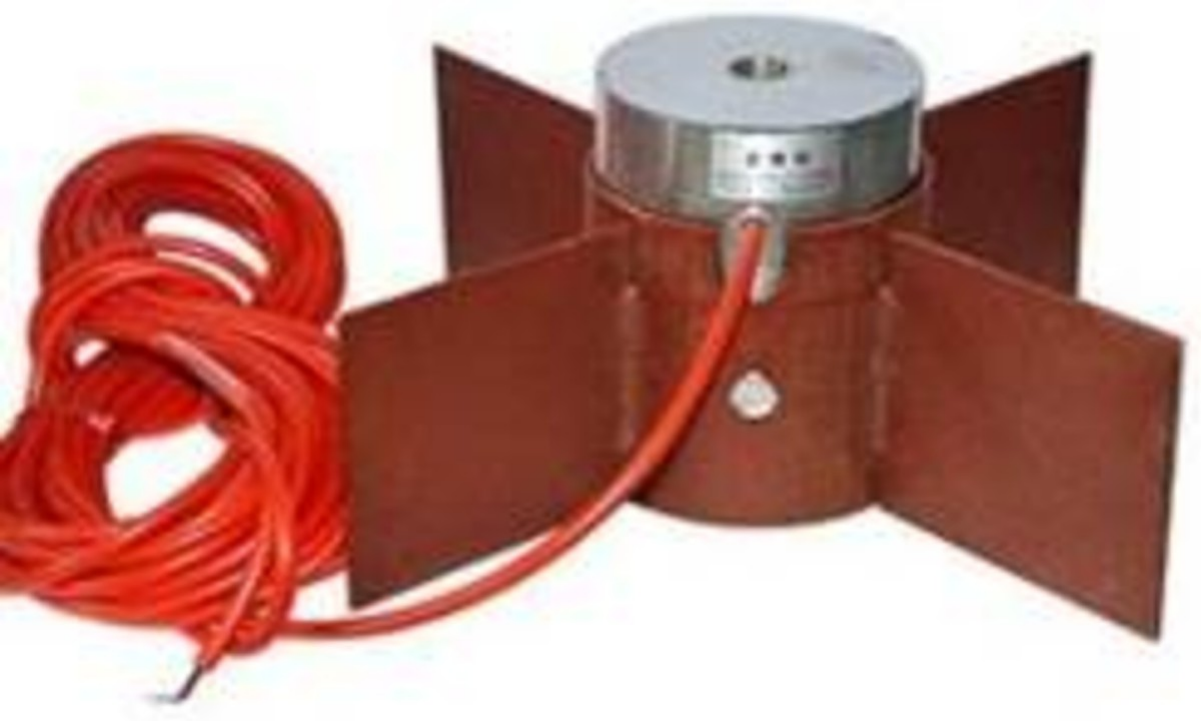
Steel bracing axial force monitoring sensor.

The steel bracing is mainly composed of φ800 and φ609 steel tubes. The monitoring sensor of the steel bracing axial force is shown in Fig 1: the axial force change data of the steel bracing is read by installing an axial force meter at the end of the steel bracing.

The axial dynamometer is equipped with an installation frame, one end of the installation frame is slotted, the other end is complete, the whole is cylindrical, the outer wall of the cylinder has four wings for welding point fixing. When installing, an electric welding machine is used for welding. In specific construction, on the basis of ensuring that the central axis of steel bracing and the center of the installation part are aligned, the two steel plates on the whole section and the supporting movable joint head are welded to ensure the firm position of the contact point.

After standing for a period of time, when the temperature of the welding part drops to an appropriate state, the axial dynamometer is placed in the mounting frame that has completed the welding operation, and then the bolts are integrated firmly.

After the steel bracing lifting procedure is finished, a steel plate with a specification of 250×250×25mm can be laid to effectively confirm that the forces between the axial force gauge and the wall steel plates play a role in a uniform state. This can also avoid the squeezing or even falling into the wall due to the small force surface of the sensor after the steel bracing is stressed, which affects the final data results.

Before applying prestress, the initial frequency of the installed axial dynamometer should be measured and recorded to ensure no damage.

Then steel is employed to support the prestress after the value reaches the design standard.

Steel bracing axial force should be measured following the monitoring requirements, and data should be read at the same time every day as far as possible to reduce the error caused by temperature.

### 2.3 Sensor measurement

**Fig 2:**
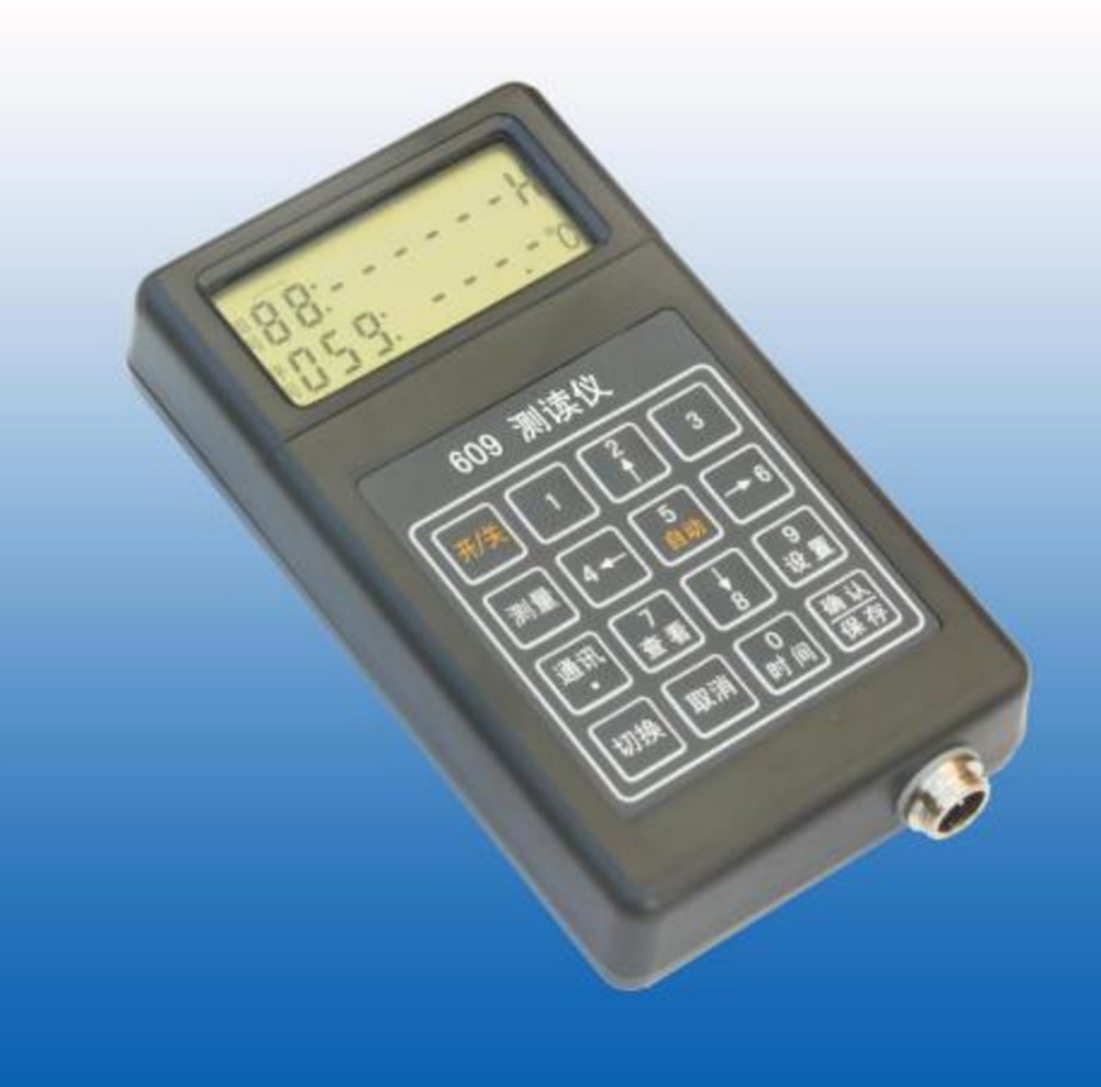
609 frequency readout.

The measurement method of vibrating string sensor is mainly measured by frequency readout, as shown in Fig 2. It exhibits the current mainstream type 609 readout. An automatic acquisition module is also used for measurement at present.

### 2.4 Calculation of steel bracing axial force

Using an axial dynamometer, steel bracing data is calculated as: $P=K(f_{1}^{2}-f_{0}^{2})$

Where, *P* is the supporting axial force (*kN*);

*K* is the sensitivity coefficient of the axial dynamometer (*kN*/*Hz*^2^);

*f*_1_ is the axial dynamometer measurement frequency value;

*f*_0_ is the initial frequency value of the axial dynamometer.

### 2.5 Monitoring results analysis

**Fig 3:**
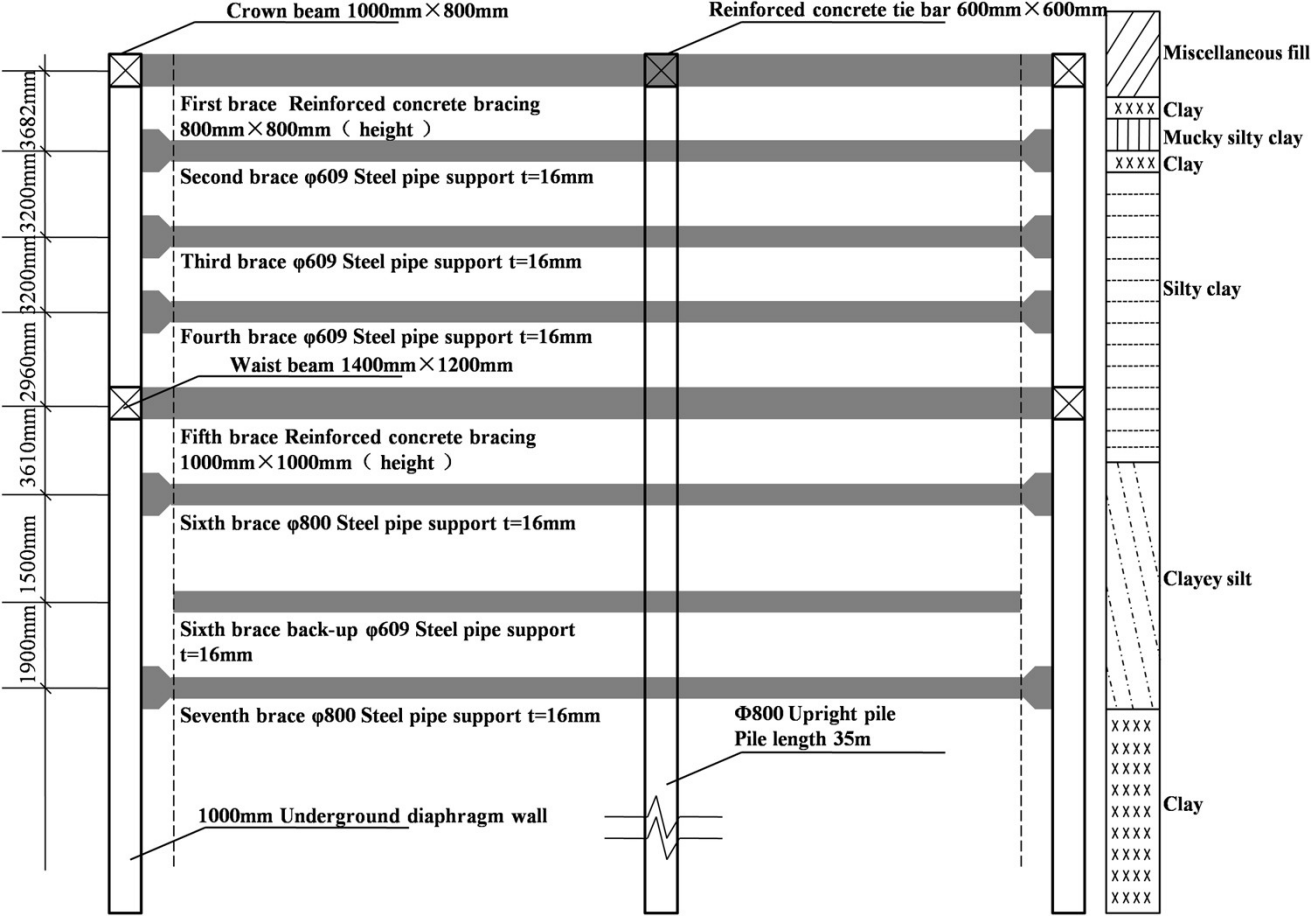
Cross-section drawing of the main envelope.

The axial force monitoring data of the steel bracing in the 5–4 foundation pit section of TJ4008 of Ningbo Rail Transit Lines 4 and 5 were selected for analysis, with the focus on the data from the erection of steel bracing in this road to the erection of the next steel bracing. This section is the transfer station section of Line 4 and Line 5. The foundation pit bracing system of the station is composed of concrete bracing and steel bracing, among which the first and fifth supports are concrete and the rest are steel. The transverse section of the main envelope structure is illustrated in Fig 3.

The standard values of each axial force of the main envelope are presented in Table 1.

**Table 1:** Standard bracing axial force values of the main envelope.

Bracing	Standard value of bracing axial /kN	Pre axial force/kN	Alarm value/kN
First bracing (concrete)	1258	-	1066
Second bracing (steel)	1996	1398	1597
Third bracing (steel)	2607	1825	2085
Fourth bracing (steel)	2671	1869	2136
Fifth bracing (concrete)	6131	-	4904
Six bracing (steel)	3851	2696	3081
Seven bracing (steel)	3926	2748	3141
Replacing bracing (steel)	2016	1411	1613

**Fig 4:**
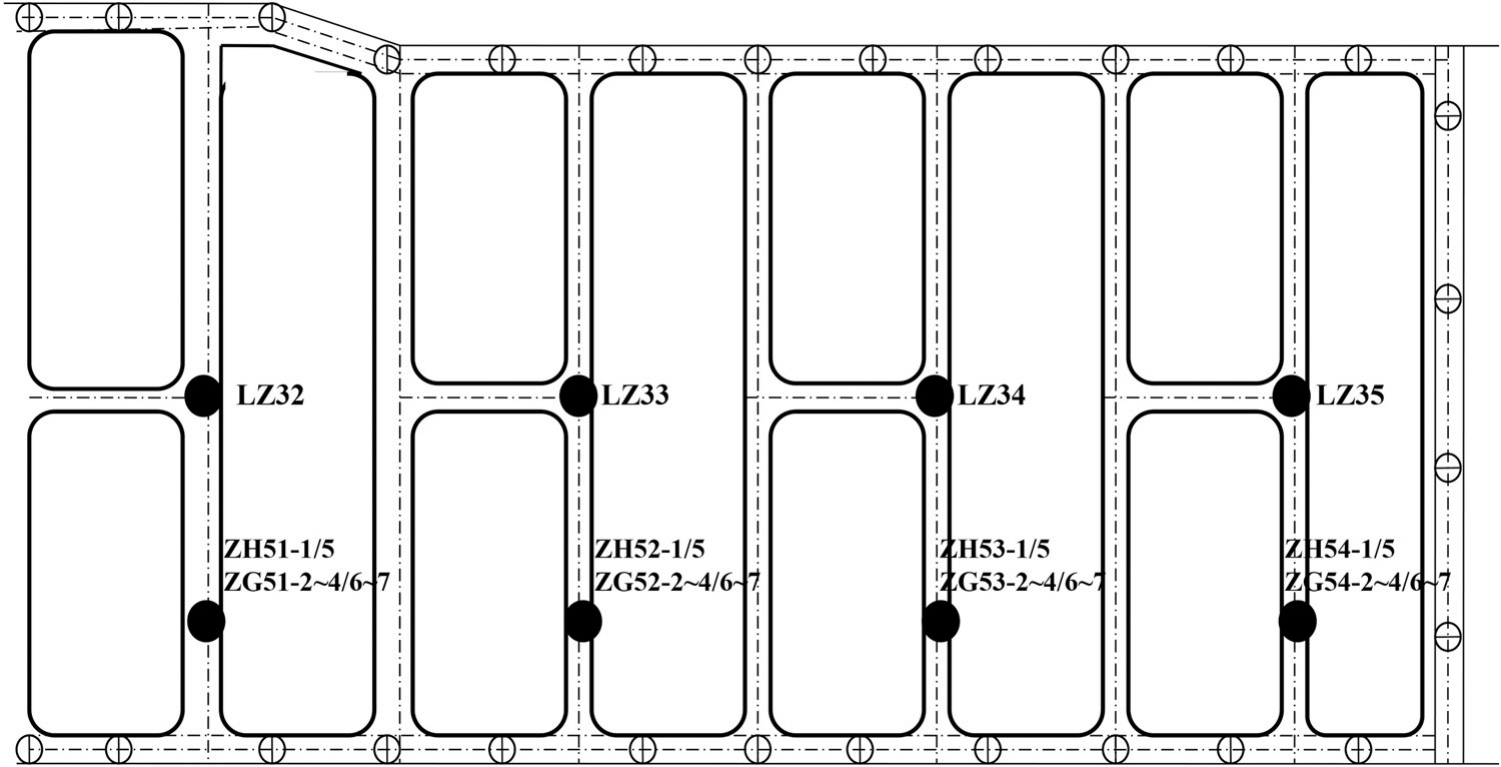
Axial force distribution of steel bracings.

The distribution of the steel bracing axial force meter is exhibited in Fig 4.

**Fig 5:**
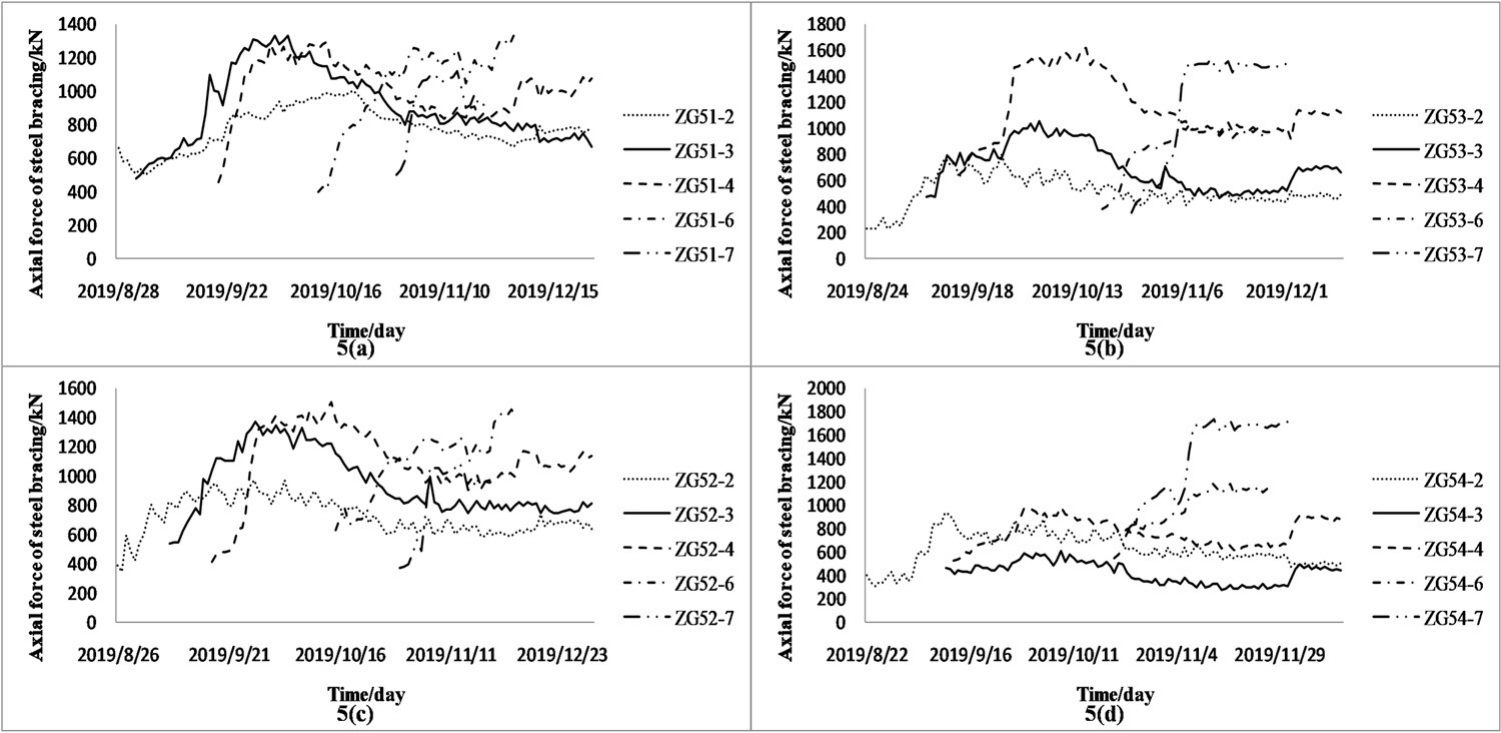
a-d. ZG54 group 2–4 and 6–7 support axial force.

Four groups of representative steel bracing axial forces with complete monitoring data were monitored and analyzed. The full change process curve of the steel bracing axial forces is displayed in Fig 5A–5D.

**Fig 6:**
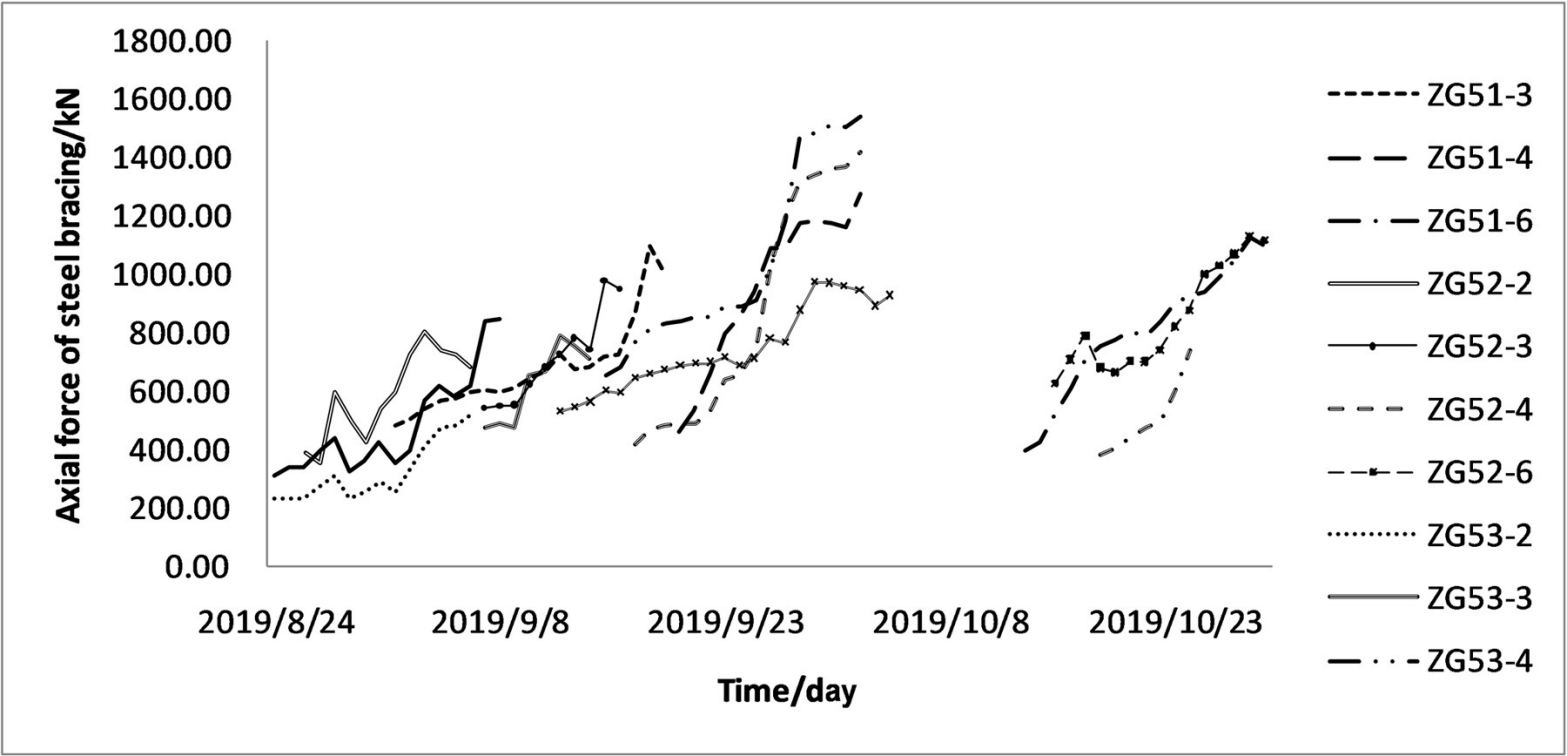
Variation of axial force in the early stage after steel bracing erection.

As comprehensively observed in Fig 5A–5D, the steel bracing has a trend of increasing first and then decreasing in the beginning with the increase of the excavation depth. However, the decrease and increase rate of each bracing axial forces data are not the same because an outward force on the envelope structure resulting from the initial axial prestressing of the steel bracing offsets some of the axial force and decreases the axial force. With the advancement of the construction, the bracing axial force increases rapidly after the foundation pit is excavated. Before the erection of the next steel bracing, the changing curve of the axial force of the current steel bracing does not increase monotonically but presents a process of fluctuating upward (Fig 6). This can be explained from two perspectives: 1) the external environment, including construction, temperature change, and support rod force; 2) the soil and wall, in which the supporting axial force generally decreases with the increase in the soil strength and increases with the decrease in the wall stiffness [21, 22]. At the later stage, the axial force of steel bracing tends to be stable, and the relationship between the axial force and time presents an “S” shaped curve, consistent with the characteristics of the Verhulst model [23]. The object of this study is the change in the current axial force of the steel bracing before the erection of the next steel bracing. Besides, the initial decrease of the axial force value is excluded. Then, the overall trend is constantly increasing and eventually tends to be stable, though the axial force of the steel bracing shows certain fluctuations. On this basis, the most basic requirements of the Verhulst model can be satisfied, and the original data can be processed for further modeling.

## 3. Comparison of data prediction models of steel bracing system

Due to the short erection time between some steel bracings and a small probability of possible deformation, there is no need for long-term monitoring, prediction, and prevention and control. However, risks are more likely to occur between two steel bracings with long intervals. Therefore, multiple sets of axial force data measured at the steel bracing monitoring point with a long-time interval between the two steel bracing erections were selected for prediction. The two groups of typical steel bracings meeting the conditions are ZG51-3 and ZG53-4. ZG51-3 and ZG53-4 aforementioned refer to different measuring points. ZG51-3 is taken as an example; ZG51 represents the first group of 5–4 steel supports of foundation pit, and -3 represents the third support (i.e., the second steel bracing). With the purpose of eliminating the influence of temperature as much as possible, the monitoring time was 7 a.m., and other influencing factors were regarded as gray quantity, such as the impact of rainfall, surrounding large machinery vibration and so on. The 10-day axial force data of the two groups of steel bracing are presented in Table 2. After the monitoring data of the axial force of the steel bracing were obtained, the monitoring data were inspected and processed. During the foundation pit excavation, the data between the two bracing erections is actually only 8–15 days. The comparison of data of different lengths shows no significant difference. Too few data prove that the excavation of the foundation pit has not been continued and there is no response risk. If the time is too long, the next support has been erected. Therefore, 10 data lengths are taken as representatives for comparison.

**Table 2:** Axial force data of steel bracing for 10 days.

ZG51-3		ZG53-4	
Time	Axial force of steel bracing/kN	Time	Axial force of steel bracing/kN
2019/09/01	481.52	2019/09/15	655.23
2019/09/02	503.09	2019/09/16	680.16
2019/09/03	537.10	2019/09/17	767.52
2019/09/04	566.00	2019/09/18	821.69
2019/09/05	576.94	2019/09/19	829.53
2019/09/06	596.29	2019/09/20	842.89
2019/09/07	601.74	2019/09/21	852.54
2019/09/08	593.81	2019/09/22	855.76
2019/09/09	607.69	2019/09/23	892.43
2019/09/10	645.73	2019/09/24	892.88

### 3.1 Calculation of the data-level ratio

Before using the GM (1, 1) model, the basic data should be inspected and transformed to test the feasibility of establishing the GM (1, 1) model. According to the gray theory of professor Deng Julong [24], the closer the data-level ratio to zero, the higher the precision of the GM (1, 1) model, and the more accurate the prediction of the axial force data of the steel bracing. The purpose of the data-level ratio test is to determine whether the sequence of the original data of the steel bracing axial force has some suitable laws, so as to judge whether a satisfactory model can be obtained. However, the calculation of the data-level ratio is actually ignored in the actual processing of many engineering data, resulting in a large distortion of model values and a great impact on the prediction results of the steel bracing.

The original sequence of numbers is set as *x*^(0)^ = (*x*^(0)^(1),*x*^(0)^(2),⋯,*x*^(0)^(*n*)). The calculation level ratio of the original sequence of numbers is:

$$\lambda^{\left(0\right)}(k)=\frac{x^{\left(0\right)}(k-1)}{x^{\left(0\right)}(k)},\;k=2,3,\cdots ,n$$ (1)

The sequence of level ratio is obtained as:

$$\lambda^{\left(0\right)}(k)=\left(\lambda^{\left(0\right)}(2),\;\lambda^{\left(0\right)}(3),\cdots ,\lambda^{\left(0\right)}(n)\right)$$ (2)

According to Formula (1), the level ration test is conducted to the original data of the steel bracing axial force. The obtained data are provided in Table 3.

**Table 3:** Raw data level ratio.

Point number	Level ratio								
ZG51-3	0.9571	0.9367	0.9489	0.9810	0.9675	0.9909	1.0134	0.9772	0.9411
ZG53-4	0.9633	0.8862	0.9341	0.9905	0.9842	0.9887	0.9962	0.9589	0.9995

### 3.2 Determination of tolerable coverage range

If it is $\lambda^{\left(0\right)}(k)\in (e^{-\frac{2}{n+1}},e^{\frac{2}{n+1}})$, the level ratio falls in the optimal interval. Thus, *x*^(0)^ meets the conditions for model establishment [24]. The covering range $X=(e^{-\frac{2}{n+1}},e^{\frac{2}{n+1}})=(0.8338,1.1994)$ can be calculated from the table. Besides, the above level ratio falls within the coverage range, satisfying the conditions for the establishment of the GM (1,1) model. No translation transformation is required. Since the Verhulst model is a power model of GM (1,1), the level ratio of the Verhulst model also follows this principle.

If not all values of *λ*^(0)^(*k*) fall with in $X=(e^{-\frac{2}{n+1}},e^{\frac{2}{n+1}})$, the original sequence must be processed and transformed. The transformation methods include logarithmic transformation, root transformation, and translation transformation. Currently, the commonly used transformation method is translation transformation:

$$y^{\left(0\right)}(k)=x^{\left(0\right)}(k)+c,\;k=1,2,\cdots ,n$$ (3)

An appropriate value *c* was added to make all level ratios of the data column fall within the covering range.

### 3.3 Comparison between gray GM (1,1) model and verhulst model

Suppose that the original observed data of the axial force of the steel bracing is:

$$x^{\left(0\right)}=\left(x^{\left(0\right)}(1),\;x^{\left(0\right)}(2),\cdots ,x^{\left(0\right)}(n)\right)$$ (4)

If the above modeling conditions are satisfied, the 1-AGO sequence is generated by a one-time accumulation:

$$x^{\left(1\right)}=\left(x^{\left(1\right)}(1),\;x^{\left(1\right)}(2),\cdots ,x^{\left(1\right)}(n)\right)$$ (5)

$x^{\left(1\right)}(k)=\sum_{i=1}^{k}x^{\left(0\right)}(i)$ in Formula (5).

*z*^(1)^(*k*) is set to denote the sequence of numbers generated by the adjacent value of *x*^(1)^, that is, *z*^(1)^(*k*) = *ax*^(1)^(*k*)+(1−*a*)*x*^(1)^(*k*−1), where *a* is 1/2. Then, the following can be obtained:

$$z^{\left(1\right)}(k)=[x^{\left(1\right)}(k)+x^{\left(1\right)}(k-1)]/2$$ (6)

The first-order differential equation of Formula (5) is established:

$$\frac{dx^{\left(1\right)}}{dt}+\hat{a}x^{\left(1\right)}=\hat{\mu }$$ (7)

Where $\hat{\mu }$ represents the relationship of data change and is the gray action quantity; $\hat{a}$ indicates the development trend of the system and is the development coefficient. The least-square method is adopted to obtain the following formula:

$$A={\left[\hat{a}\hat{\mu }\right]}^{T}={\left(B^{T}B\right)}^{-1}B^{T}Y_{n}$$ (8)

where, for the GM (1,1) model, there are $B=[\begin{array}{cc} -Z^{\left(1\right)}(2) & 1\\ -Z^{\left(1\right)}(3) & 1\\ -Z^{\left(1\right)}(4) & 1\\ \vdots & \vdots \\ -Z^{\left(1\right)}(n) & 1 \end{array}],\;Y_{n}=[\begin{array}{c} x^{\left(0\right)}(2)\\ x^{\left(0\right)}(3)\\ x^{\left(0\right)}(4)\\ \vdots \\ x^{\left(0\right)}(n) \end{array}]$

For the Verhulst model, there are $B=[\begin{array}{cc} -z^{\left(1\right)}(2) & {\left(z^{\left(1\right)}(2)\right)}^{2}\\ -z^{\left(1\right)}(3) & {\left(z^{\left(1\right)}(3)\right)}^{2}\\ -z^{\left(1\right)}(4) & {\left(z^{\left(1\right)}(4)\right)}^{2}\\ \vdots & \vdots \\ -z^{\left(1\right)}(n) & {\left(z^{\left(1\right)}(n)\right)}^{2} \end{array}],\;Y_{n}=[\begin{array}{c} x^{\left(0\right)}(2)\\ x^{\left(0\right)}(3)\\ x^{\left(0\right)}(4)\\ \vdots \\ x^{\left(0\right)}(n) \end{array}]$.

The values of $\hat{\mu },\hat{a}$ in A are substituted into Formula (7) to obtain the GM (1,1) model:

$${\hat{x}}^{\left(1\right)}(k+1)=\left(x^{\left(0\right)}(1)-\frac{\hat{\mu }}{\hat{a}}\right)e^{-\hat{a}k}+\frac{\hat{\mu }}{\hat{a}}$$ (9)

The Verhulst model:

$${\hat{x}}^{\left(1\right)}(k+1)=\frac{1}{(\frac{1}{x^{\left(0\right)}(1)}-\frac{\hat{\mu }}{\hat{a}})e^{\hat{a}k}+\frac{\hat{\mu }}{\hat{a}}}$$ (10)

Where *k* = 1,2,3,⋯,*n*−1.

The simulated data reduced can be generated by the accumulative reduction of ${\hat{x}}^{\left(1\right)}(k+1)$:

$${\hat{x}}^{\left(0\right)}(k+1)={\hat{x}}^{\left(1\right)}(k+1)-{\hat{x}}^{\left(1\right)}(k)$$ (11)

After the prediction model is established, the accuracy of the prediction model should be tested to determine whether it can be used to predict the axial force of steel bracing. Besides, a posteriori test is generally performed to examine the accuracy of the predictive model. A posteriori difference test is conducted to compare the data obtained by the model with the obtained historical data.

The average value of the original observed data *x*^(0)^(*k*), *k* = 1,2,⋯,*n* is:

$$\bar{x}=\frac{1}{n}\sum_{k=1}^{n}x^{\left(0\right)}(k)$$ (12)

The mean value of the residual is:

$$\bar{e}=\frac{1}{n}\sum_{k=1}^{n}e(k)$$ (13)

The variance of the original data is:

$${S_{1}}^{2}=\frac{1}{n}\sum_{k=1}^{n}{\left(x^{\left(0\right)}(k)-\bar{x}\right)}^{2}$$ (14)

The variance of the residual is:

$${S_{2}}^{2}=\frac{1}{n}\sum_{k=1}^{n}{\left(e(k)-\bar{e}\right)}^{2}$$ (15)

The posterior difference ratio is:

$$C=\frac{S_{2}}{S_{1}}$$ (16)

The frequency of small errors is:

$$P=P\{|e(k)-\bar{e}|<0.6745S_{1}\}$$ (17)

The larger the value of *S*_1_, the greater the dispersion of the original data of the steel supporting axial force. The greater the value of *S*_2_, the greater the dispersion of the residual. Therefore, the smaller the posterior difference ratio, the smaller the dispersion between the simulated value and the actual value, and the higher the accuracy of the model. Moreover, the greater the frequency of small error *P*, the more the points whose residual and mean have a difference of less than 0.6745*S*_1_.

According to Table 4, the accuracy of the model can be demonstrated.

**Table 4:** Evaluation of model accuracy.

Accuracy grade	*P*	*C*
Grade 1 (Good)	>0.95	<0.35
Grade 2 (Qualified)	>0.8	<0.5
Grade 3 (General)	>0.7	<0.65
Grade 4 (Unqualified)	≤0.7	≥0.65

MATLAB was used to establish a gray prediction model for the monitoring data according to Eqs (4)–(11). The above results were tested following Eqs (12)–(17) to obtain the results, as presented in Tables 5 and 6:

**Table 5:** ZG51-3 grey prediction model results.

Date	Actual value/kN	GM (1,1) Model			Verhulst Model		
		Model value/kN	Residual	Relative error/%	Model value/kN	Residual	Relative error/%
2019/9/1	481.52	481.52	0.00	0.00	481.52	0.00	0.00
2019/9/2	503.09	525.67	22.58	4.49	505.69	2.60	0.52
2019/9/3	537.1	538.70	1.60	0.30	528.45	-8.65	-1.61
2019/9/4	566	552.06	-13.94	-2.46	549.67	-16.33	-2.89
2019/9/5	576.94	565.75	-11.19	-1.94	569.28	-7.66	-1.33
2019/9/6	596.29	579.77	-16.52	-2.77	587.25	-9.04	-1.52
2019/9/7	601.74	594.14	-7.60	-1.26	603.60	1.86	0.31
2019/9/8	593.81	608.87	15.06	2.54	618.37	24.56	4.14
2019/9/9	607.69	623.97	16.28	2.68	631.63	23.94	3.94
2019/9/10	645.73	639.44	-6.29	-0.97	643.47	-2.26	-0.35
C		0.27			0.27		
P		1			1		

**Table 6:** ZG53-4 grey prediction model results.

Date	Actual value/kN	GM (1,1) Model			Verhulst Model		
		Model value/kN	Residual	Relative error/%	Model value/kN	Residual	Relative error/%
2019/9/15	655.23	655.23	0.00	0.00	655.23	0.00	0.00
2019/9/16	680.16	742.97	62.81	9.23	709.57	29.41	4.32
2019/9/17	767.52	762.53	-4.99	-0.65	755.19	-12.33	-1.61
2019/9/18	821.69	782.60	-39.09	-4.76	792.30	-29.39	-3.58
2019/9/19	829.53	803.20	-26.33	-3.17	821.71	-7.82	-0.94
2019/9/20	842.89	824.34	-18.54	-2.20	844.55	1.66	0.20
2019/9/21	852.54	846.04	-6.50	-0.76	862.00	9.46	1.11
2019/9/22	855.76	868.31	12.55	1.47	875.18	19.42	2.27
2019/9/23	892.43	891.17	-1.25	-0.14	885.03	-7.40	-0.83
2019/9/24	892.88	914.63	21.75	2.44	892.35	-0.53	-0.06
C		0.34			0.20		
P		0.9			1		

The prediction equations corresponding to the GM (1,1) model are:

ZG51-3: ${\hat{x}}^{\left(1\right)}(k+1)=21204.33e^{0.024488k}-20722.81$;

ZG53-4: ${\hat{x}}^{\left(1\right)}(k+1)=28224.36e^{0.025983k}-27569.13$.

The prediction equations corresponding to the Verhulst model are:

ZG51-3: ${\hat{x}}^{\left(1\right)}(k+1)=\frac{1}{0.000699e^{-0.153267k}+0.001378}$;

ZG53-4: ${\hat{x}}^{\left(1\right)}(k+1)=\frac{1}{0.00043e^{-0.316822k}+0.001096}$.

**Fig 7:**
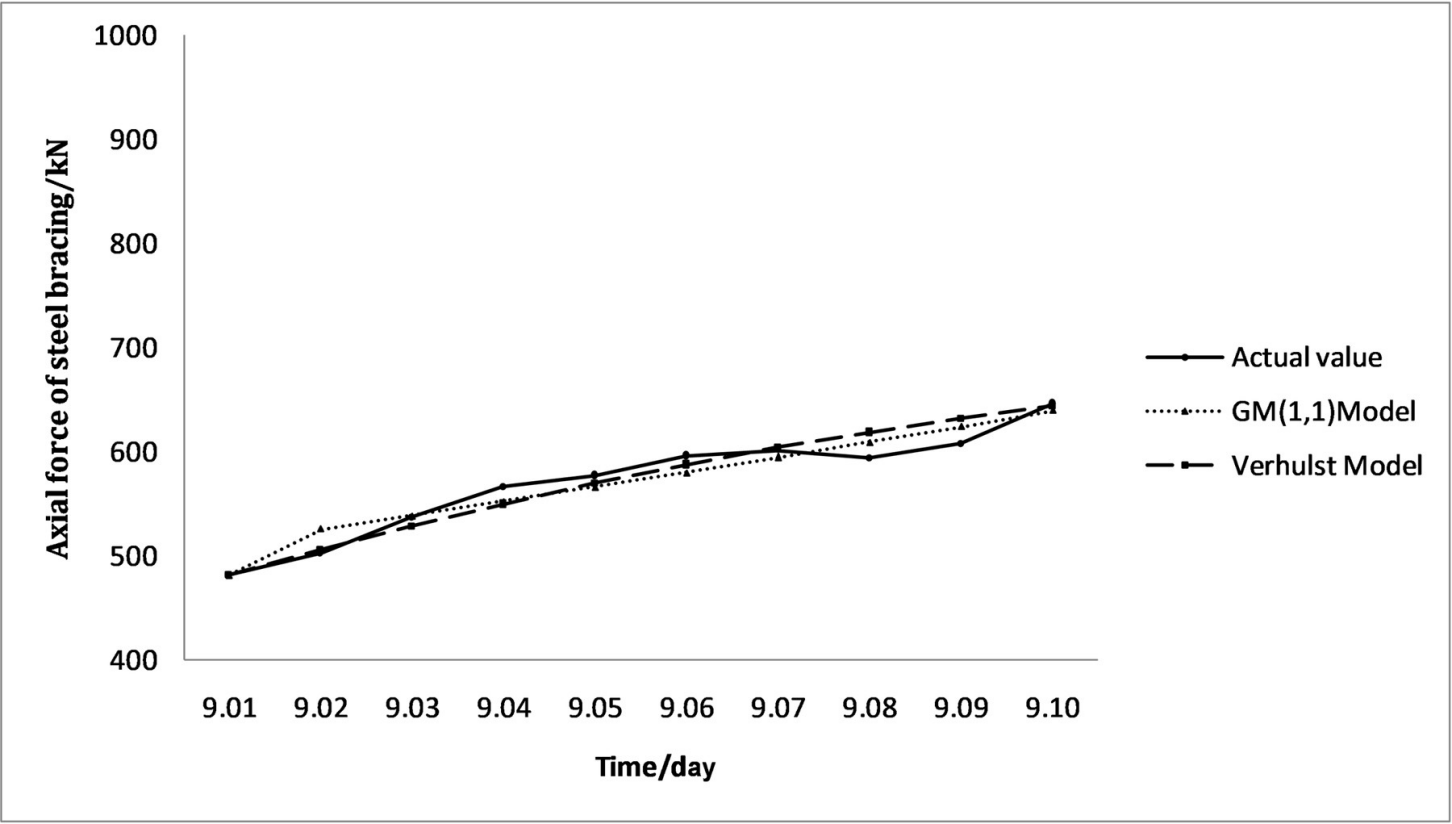
Comparison of ZG51-3 the actual value and the model value.

**Fig 8:**
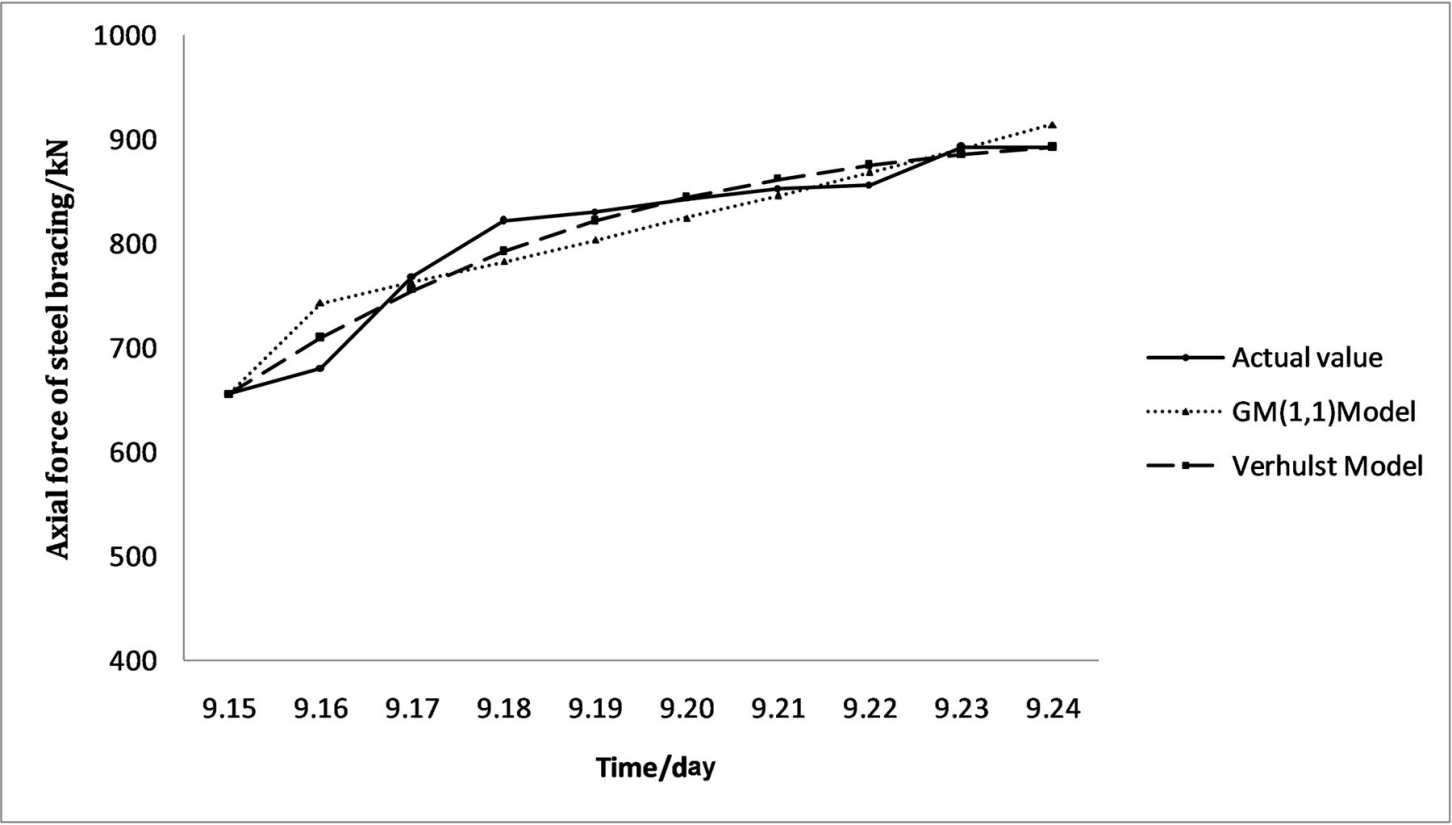
Comparison of ZG53-4 the actual value and the model value.

The corresponding comparison results are illustrated in Figs 7 and 8. The above residual value, relative error, and accuracy results demonstrate that the predicted value of the Verhulst model is more consistent with the actual value and has a better smoothness.

It can be comprehensively judged that the gray prediction model can be employed to predict the short-term data from the erection of this steel bracing to the erection of the next one, and the Verhulst model has better accuracy than the GM (1,1) model.

In addition to the influence of data dispersion and residual value dispersion, the influence factors of functional transformation should also be considered for the accuracy of the model. The smoothness and reduction accuracy need to be comprehensively considered to further improve the accuracy of the model, enabling the modeling accuracy to reach the optimal [25]. During the process of predicting the axial force of steel bracing, the further optimization of the Verhulst model will yield half the result with twice the effort on the premise that the accuracy of the conventional modeling conforms to the accuracy evaluation table of the model, owing to the complex situation of the actual construction site and the requirement of the erection time of steel bracing.

## 4. Safety forewarning model of steel bracing system

In the safety evaluation of various structures of the foundation pit, different evaluation methods are selected to meet the personalized needs of various monitoring indicators [26]. Considering the uncertainty and diversity of factors affecting the steel bracing, the evaluation criteria of the confidence interval method are utilized to describe and reflect the evaluation indexes more scientifically, making the safety and stability analysis of the steel bracing system more comprehensive. Generally, the establishment of a safety evaluation model includes the establishment of safety indicators, the establishment of a safety evaluation model, and the classification of safety early forewarning levels [27].

The main monitoring index of the steel bracing structure is the bracing axial force, and the safety control index of the axial force is the safety limit value specified by the design of the steel bracing. The operation of the steel bracing structure is safe if the measured value is within the numerical range of the design monitoring index.

### 4.1 Safety index formulation of steel bracing axial force

Engineering safety monitoring is one of the imperative methods to provide a construction basis, with an ultimate goal of conducting safety monitoring and judgment on the stability of the engineering. Given the uncertainty and particularity of engineering itself, it is very difficult to formulate the evaluation standards, though the evaluation methods and standards of engineering safety monitoring are clear. At present, engineering safety monitoring is mainly divided into two categories (using the comprehensive evaluation method and formulating the safety monitoring index of the physical quantity of engineering monitoring) to measure whether the engineering operation is safe.

Safety monitoring indicators can be divided into two categories: 1) the forewarning value, which can judge whether the engineering is running normally or not; 2) the danger value, which can judge whether the engineering is running safely or not. Currently, there are many methods to formulate safety monitoring indicators, such as the mathematical model method, ultimate strength analysis method, safety factor method, kernel density estimation theory, cloud model, and comprehensive formulation method [28–32].

Regarding the formulating deformation monitoring indicators, its maximum bearing capacity is calculated based on the ability of the steel bracing to withstand the load to obtain the forewarning value and danger value corresponding to the monitoring effect under the load. However, it is relatively complicated to formulate the deformation safety indicators considering that the steel bracing might not meet the maximum load and the bearing capacity of the steel bracing is constantly changing. Therefore, only the confidence interval estimation method is used for the calculation to obtain the values of the monitoring indicators under the mathematical method [33].

First, suitable statistics and comprehensive analysis methods are selected to establish the corresponding mathematical model of deformation on the basis of comprehensively analyzing the engineering practice; second, the confidence belt range (*ε* = ±*ns*) is set; third, the measured value *δ*(*t*) is compared with the corresponding model value *Y*(*t*). Specifically, if the difference *d* is within the allowed tolerance range, the steel bracing runs normally; otherwise, it runs abnormally. The deformation monitoring model is:

Y(t)=δ(t)±ns (18)

Where *s* denotes the residual standard deviation; *n* represents the confidence coefficient related to the significance level and the sample size, *α* = 5%,*n* = 1.96; *δ*(*t*) indicates the deformation mathematical model.

### 4.2 Confidence interval estimation method

If the significance level *α*(generally 1%-5%) is taken, *P*_*α*_ = *α* is a small probability event. If this situation occurs, it can be preliminarily identified that there is an anomaly. The focus in the specific determination is the past observed data of the steel bracing. Besides, a mathematical model is established through regression analysis and other effective ways. The difference ($E-\hat{E}$) between the monitoring effect size $\hat{E}$ and the measured value is solved. Additionally, if the probability that this value is within the scope of the confidence belt (Δ = *iσ*) is 100(1−*α*)%, the steel bracing runs normally; otherwise, it runs abnormally [34]. Regarding the previous observation data, the residual obtained by the gray prediction model of the steel bracing axial force mentioned above is used as the calculation standard, and the last observation day is taken as the evaluation standard.

At this time, the monitoring indicators of the monitoring effect size are:

$$E_{m}=E\pm \Delta$$ (19)

For the safety evaluation model of confidence interval estimation, the interval estimation of normal population mean (*t* distribution; *σ*^2^ is unknown) can be adopted to solve the confidence interval of *μ*.

Suppose the totality is *ξ*~*N*(*μ*, *σ*^2^), where *μ* and *σ*^2^ are unknown. Suppose a sample of *ξ* is (*ξ*_1_, *ξ*_2_,⋯,*ξ*_*n*_), the 1−*α* confidence interval of *μ* is solved. It suggests that the random interval (*T*_1_(*ξ*_1_, *ξ*_2_,⋯,*ξ*_*n*_), *T*_2_(*ξ*_1_, *ξ*_2_,⋯,*ξ*_*n*_) is found to allow *P*{*T*_1_<*θ*<*T*_2_} = 1−*α*) to hold, so as to construct a sample function with known distribution.

According to $\xi ∼(\mu ,\frac{\sigma^{2}}{n})$, the following formula can be obtained:

$$t=\frac{\bar{\xi }-\mu }{S^{*}/\sqrt{n}}∼t(n-1)$$ (20)

Following the Auantile Theory, the following formula can be obtained:

$$P\left\{|t|<t_{1-\frac{\alpha }{2}}(n-1)\right\}=1-\alpha$$ (21)

That is:

$$P\left\{|\frac{\bar{\xi }-\mu }{S^{*}/\sqrt{n}}|<t_{1-\frac{\alpha }{2}}(n-1)\right\}=1-\alpha$$ (22)

Namely:

$$P\left\{\bar{\xi }-\frac{S^{*}}{\sqrt{n}}t_{1-\frac{\alpha }{2}}(n-1)<\mu <\bar{\xi }+\frac{S^{*}}{\sqrt{n}}t_{1-\frac{\alpha }{2}}(n-1)\right\}=1-\alpha$$ (23)

Under the unknown variance, the confidence interval of the normal population mean is:

$$\bar{\xi }\pm \frac{S^{*}}{\sqrt{n}}t_{1-\frac{\alpha }{2}}(n-1)$$ (24)

Because $(n-1)S^{{*}^{2}}=nS^{2}$, the 1−*α* confidence interval of *μ* can be rewritten as:

$$\bar{\xi }\pm \frac{S}{\sqrt{n-1}}t_{1-\frac{\alpha }{2}}(n-1)$$ (25)

Afterward, the above calculation results are further analyzed based on the results of the above gray prediction model. The absolute values of the residual values in Tables 4 and 5 are calculated according to Eqs (20)–(25), with the last observation day as the evaluation standard. According to the confidence interval estimation method, the mean value and standard deviation of the gray prediction model are $\bar{e}$ and *S*_2_, respectively, which are calculated by the MATLAB code with significance levels of 5% and 2% for $E-\hat{E}$ confidence intervals. The results of the confidence interval calculation are offered in Table 7.

**Table 7:** Confidence interval calculation results.

Point location	Mean value	Standard deviation	5%confidence interval	Half-band width Δ_0.05_	2%confidence interval	Half-band width Δ_0.02_
ZG51-3	10.52	9.18	(3.04,18.00)	3.74	(1.12,19.91)	4.70
ZG53-4	12.99	10.89	(4.11,21.86)	4.44	(1.84,24.13)	5.58

### 4.3 Classification of security forewarning levels

The safety evaluation index of steel bracing deformation can be formulated using the confidence interval estimation method. In the process of monitoring the effect size, the upper and lower limits of the monitoring index can be set as *E*_*m*1_ and *E*_*m*2_, respectively. If the effect size value $\hat{E}\in (E_{m1},E_{m2})$ in the steel bracing is monitored, the steel bracing operates normally; otherwise, it is abnormal. In practice, significance levels α = 5% (corresponding to half-bandwidth Δ_0.05_) and α = 2% (corresponding to half-bandwidth Δ_0.02_) are taken as the demarcating points for the classification of security early forewarning levels.

The proposed safety forewarning levels are as follows:

**Table:** 

$\hat{E}\in [E-0.5\Delta_{0.05},E+0.5\Delta_{0.05}]$	Safe operation;
$\hat{E}\in [E-\Delta_{0.05},E-0.5\Delta_{0.05})\cup (E+0.5\Delta_{0.05},E+\Delta_{0.05}]$	Relatively safe operation;
$\hat{E}\in [E-\Delta_{0.02},E-\Delta_{0.05})\cup (E+\Delta_{0.05},E+\Delta_{0.02}]$	Relatively dangerous operation;
$\hat{E}\in [-\infty ,E-\Delta_{0.02})\cup (E+\Delta_{0.02},+\infty ]$	Dangerous operation.

According to the proposed safety forewarning levels, it is relatively safe for the steel bracing when the residual value is less than the mean value. Thus, only half of the interval is obtained, and the corresponding evaluation criteria and the absolute value of the residual value of the model on the observation day can be obtained from Table 8.

**Table 8:** Evaluation criteria table and observed daily residual value.

Interval	ZG51-3	ZG53-4
Safe operational interval	[0,14.26]	[0,17.43]
Relatively safe operational interval	(14.26, 18.00]	(17.43, 21.86]
Relatively dangerous operational interval	(18.00, 19.91]	(21.86, 24.13]
Dangerous operational interval	(19.91,+∞)	(24.13,+∞)
Absolute value of model residual value on the observation day	2.26	0.53

The comparison reveals that the residual value of the model on observation day 2.26∈[0,14.26], 0.53∈[0,17.43] is within the safe operational interval, reflecting that the two steel bracings are in a safe operating state.

### 4.4 Comprehensive evaluation of model

Concerning the safety evaluation of confidence interval estimation, the small probability principle of statistics is used to compare the actual monitoring value with the model value, so as to realize the safety evaluation of the monitoring target. In this study, Δ_0.05_ and Δ_0.02_ and their half-bandwidth data are taken as the cut-off points. This evaluation criterion has reliable engineering practical value and reflects the current operation state of steel bracing to some extent. The pre-processing of original data, the formulation of monitoring indexes, and the safety evaluation model are limited to a single physical quantity, namely, the analysis of deformation observation data. Thus, the corresponding mathematical model is established using the mathematical and mechanical methods based on the qualitative analysis of these data to evaluate the operation state of the steel bracing. However, it is unreasonable to only use the mathematical model of single measurement data for evaluation due to the complex conditions of the construction site. The detailed reasons are described as follows.

1. The working state of steel bracing is influenced by many factors, such as temperature and humidity, concrete creep, and foundation pit envelope. Besides, the discriminant index of its operating state is not the only value and should be combined with the design value and forewarning value given by the design.
2. Various factors have different effects on the safety of steel bracing. In the specific construction stage, their influence degrees are continuously changing with the constantly changing environment and the continuous promotion of the project. Therefore, the influence weight of each factor should be emphasized from multiple measurement points.
3. The influencing factors should be determined first for performing a comprehensive evaluation, such as the original site inspection and design. Nevertheless, the factors that cannot be quantified, such as concrete creep, may have some potential uncertainties.
4. The safety forewarning model divides the forewarning levels through confidence intervals on the basis of the mathematical-statistical model. Moreover, the forewarning should also be based on the physical mechanism model. For example, numerical simulation is adopted to simulate the stress transfer path and size after the removal or failure of single bracing.

To sum up, a more comprehensive safety monitoring of the steel bracing can be achieved by deeply analyzing all kinds of information and enabling the expert group with rich experience to make the final evaluation and decision in the case of abnormalities based on the safety forewarning model.

## 5. Conclusion

According to the steel bracing data of No.5-4 foundation pit in Ningbo Tail Transit Lines 4 and 5, the change law of axial force between the two steel bracings was studied by the data analysis method. Besides, the GM (1,1) model and Verhulst model were compared to predict the axial force of the steel bracing, judge its operation state, and divide the safety forewarning levels. The conclusions are drawn as follows.

1. Based on the characteristics of low sample density of steel bracing system data in foundation pit, the reasons for establishing the gray prediction model of steel bracing axial force are provided, and the feasibility of establishing the gray prediction model is established. As indicated by the corresponding engineering data and comprehensive judgment, the accuracy of the GM (1,1) model and the Verhulst model is good. It is verified that the grey prediction model can be used to predict the short-term data of axial force between two steel braces.
2. By comparing the GM (1,1) model and the Verhulst model, it can be demonstrated that the residual value of the Verhulst model is smaller, it has a more accurate C-value and P-value, and the curve presented is smoother. The above results are sufficient to confirm that the Verhulst model can be used to predict the short-term data of axial force between two steel braces with better accuracy and coincidence.
3. The safety forewarning levels of the corresponding steel bracing system were classified based on the prediction results of the Verhulst model, the proposed deformation monitoring indexes, and the safety forewarning model of confidence interval estimation. It was revealed that the steel bracing system was operating in the safety interval, which was consistent with the actual engineering situation. Thus, the method has some reliable engineering practical value.
4. The model is easy to calculate in engineering and has good applicability in the prediction and safety forewarning of steel bracing systems. Meanwhile, it can provide theoretical references for similar engineering parts, such as the prediction of concrete bracing axial force. Furthermore, the accuracy and agility of automated prediction and forewarning can be significantly improved by adding the Verhulst model prediction and confidence interval estimation safety forewarning model into the automated monitoring and data processing system.
